# Supplementary figures and images for: Large-scale identification of plasma membrane repair proteins revealed spatiotemporal cellular responses to plasma membrane damage
Source: eLife. 2026 Mar 10;14:RP108585. doi: 10.7554/eLife.108585 (PMC12975127; doi:10.7554/eLife.108585)

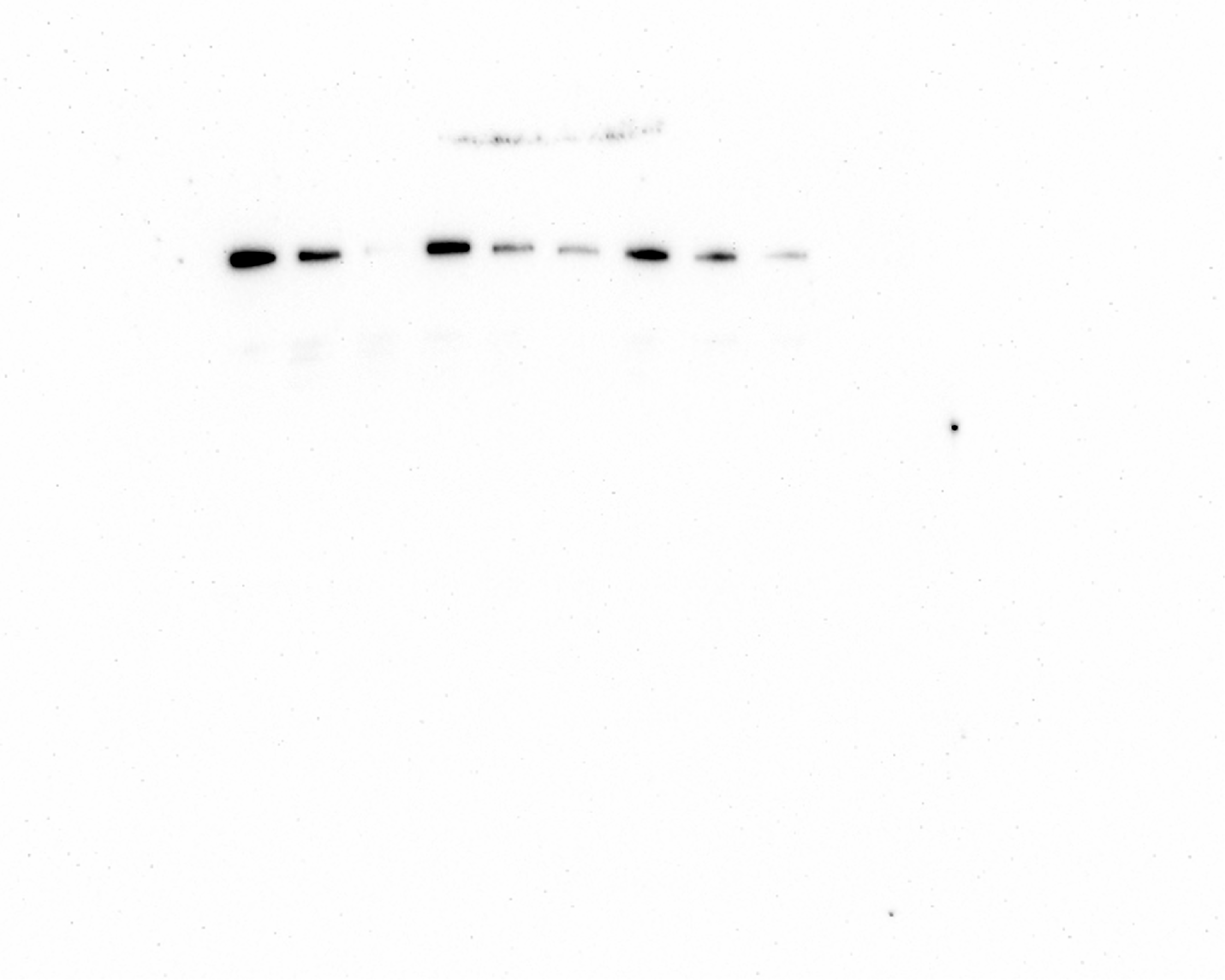

Supplement: Figure 4—figure supplement 3—source data 1. [file elife-108585-fig4-figsupp3-data1.zip › Figure 4–figure supplement 3–Source 1 Original files of western blots/Figure 4–figure supplement 3A/Myc.tif]

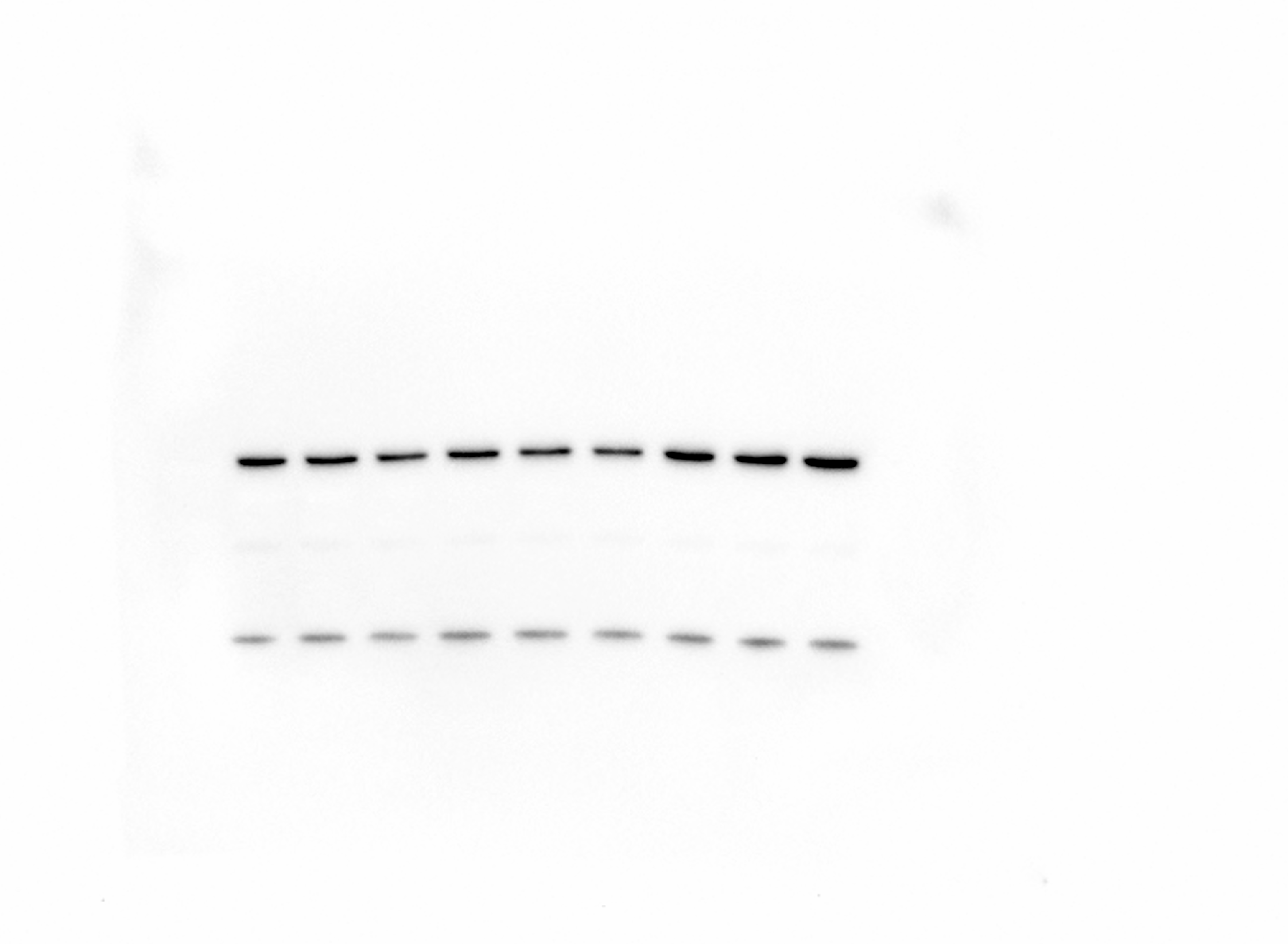

Supplement: Figure 4—figure supplement 3—source data 1. [file elife-108585-fig4-figsupp3-data1.zip › Figure 4–figure supplement 3–Source 1 Original files of western blots/Figure 4–figure supplement 3A/Tubulin.tif]

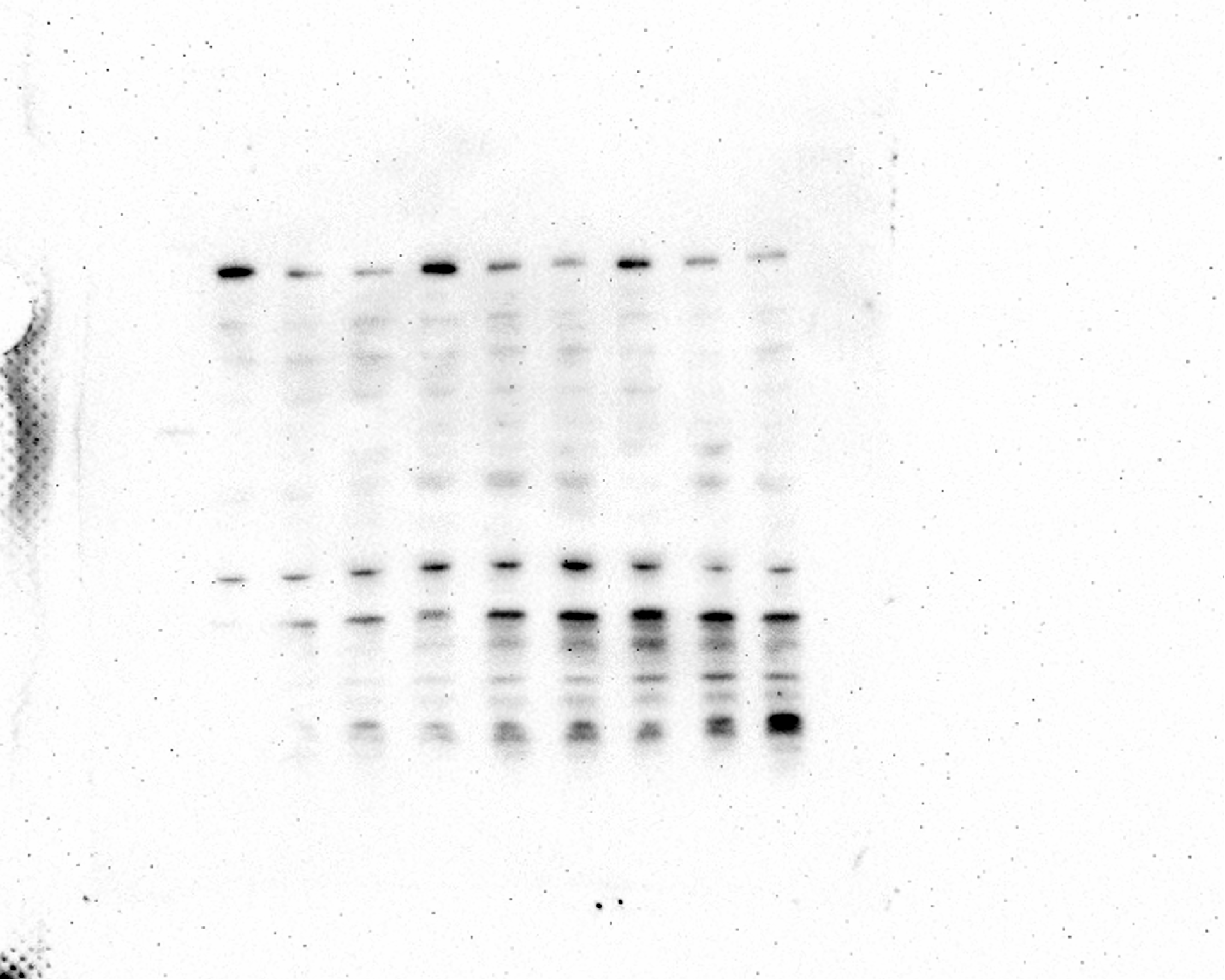

Supplement: Figure 4—figure supplement 3—source data 1. [file elife-108585-fig4-figsupp3-data1.zip › Figure 4–figure supplement 3–Source 1 Original files of western blots/Figure 4–figure supplement 3B/GFP.tif]

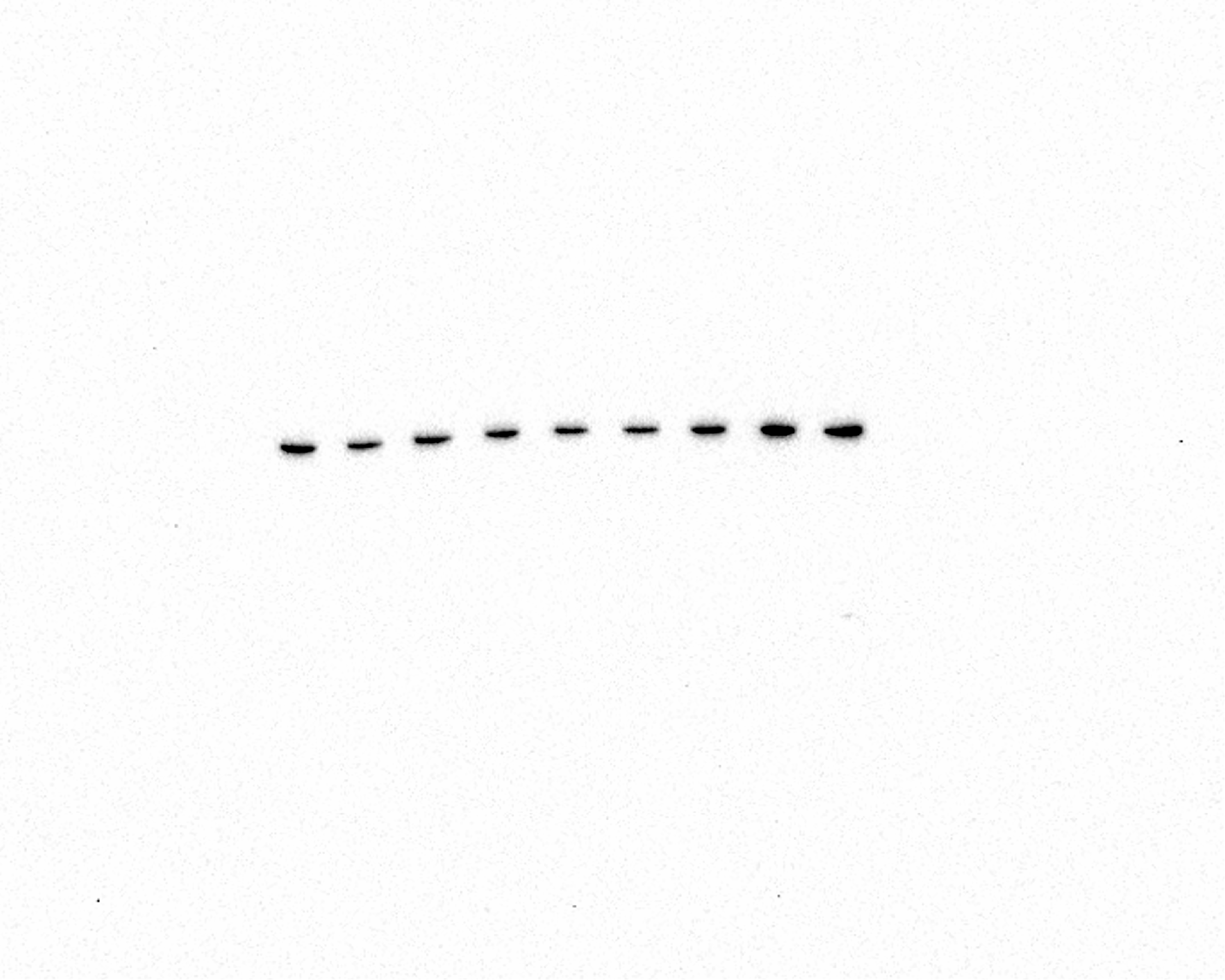

Supplement: Figure 4—figure supplement 3—source data 1. [file elife-108585-fig4-figsupp3-data1.zip › Figure 4–figure supplement 3–Source 1 Original files of western blots/Figure 4–figure supplement 3B/Tubulin.tif]

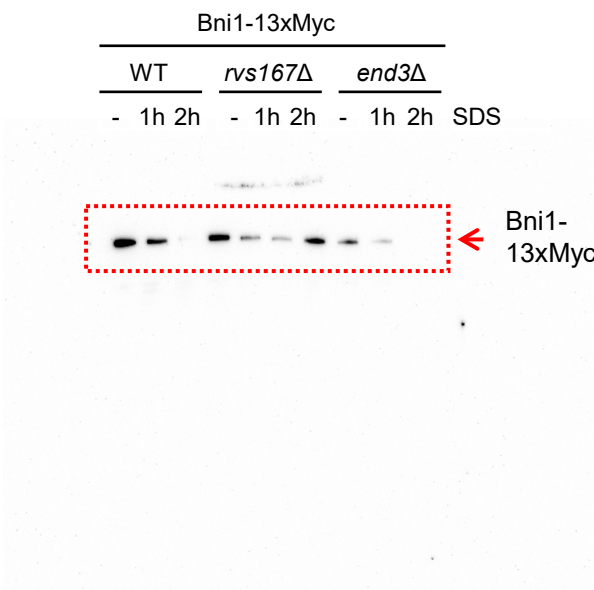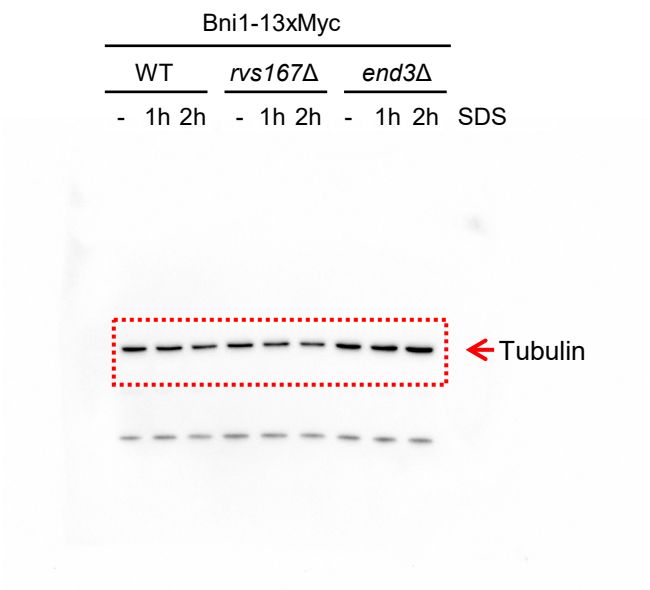

Supplement: Figure 4—figure supplement 3—source data 2. [file elife-108585-fig4-figsupp3-data2.zip › Figure 4–figure supplement 3–Source Data 2 PDF files of western blots with labels/Figure 4–figure supplement 3A.pdf]

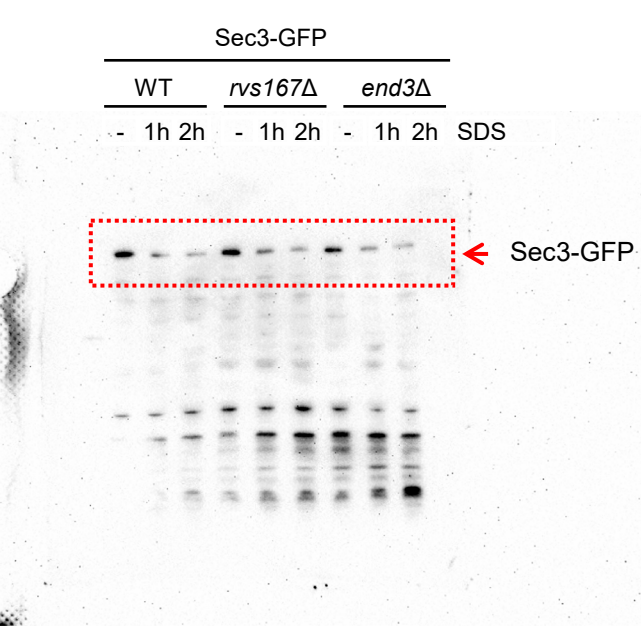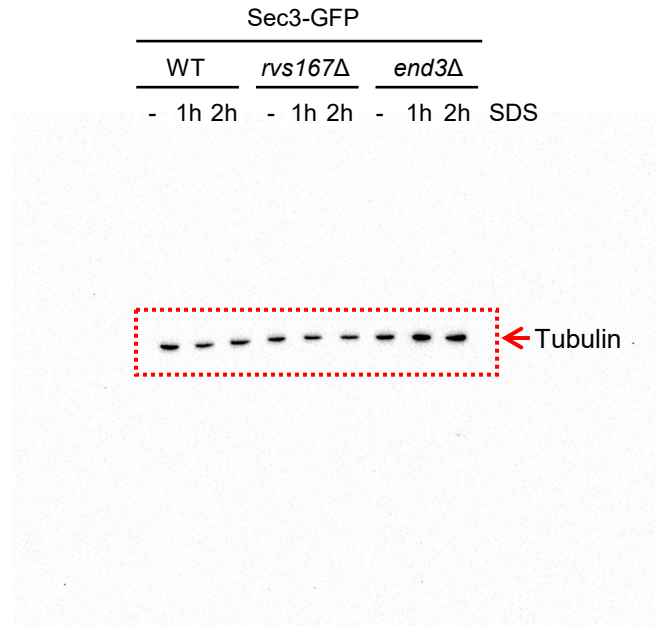

Supplement: Figure 4—figure supplement 3—source data 2. [file elife-108585-fig4-figsupp3-data2.zip › Figure 4–figure supplement 3–Source Data 2 PDF files of western blots with labels/Figure 4–figure supplement 3B.pdf]
